# Bridging implementation science and policy change: a Global South perspective

**DOI:** 10.3389/frhs.2026.1820779

**Published:** 2026-07-01

**Authors:** Adolfo Rubinstein, Vilma Irazola

**Affiliations:** 1 Center for Implementation and Innovation in Health Policies (CIIPS), Institute for Clinical Effectiveness and Health Policy, Buenos Aires, Argentina; 2 Department of Chronic Disease Research, Institute for Clinical Effectiveness and Health Policy, Buenos Aires, Argentina

**Keywords:** governance, health policy, health systems, implementation science, low- and middle-income countries, policy implementation

## Abstract

*This narrative and conceptual review examines the interface between implementation science and health policy research. Drawing on literature from both disciplines, we analyze key areas of convergence and divergence, explore how these distinctions contribute to the persistent ‘know-do gap,’ and discuss opportunities for integrated approaches connecting micro-level implementation processes with macro-level policy dynamics, with particular attention to LMICs.* Implementation science and health policy research have evolved largely in parallel, despite sharing a common objective: improving population health by translating knowledge into action. Implementation science focuses on integrating evidence-based interventions into real-world settings, while policy research examines how policies are formulated, adopted, and sustained within complex political systems. This disciplinary separation has contributed to a persistent “know–do gap,” particularly in low-and middle-income countries (LMICs), where fragmented health systems, political economy constraints, and donor influence shape both policy trajectories and implementation realities. The disconnect between researchers and policymakers often stems from differing timing, priorities, and even language. Researchers may not address the immediate concerns of policymakers, and scientific evidence alone seldom drives policy changes. In LMICs, this divide is exacerbated by political cycles, vested interests, and the significant influence of international donors. In this review, we argue that bridging implementation science and policy research is not merely desirable but necessary. We examine how differences in scope, actors, settings, and theoretical frameworks have reinforced this divide, and we propose the development of integrated, hybrid approaches that connect micro-level implementation processes with macro-level policy dynamics. Bridging the gap between implementation research and policy change necessitates a context-sensitive approach that acknowledges the complexities of political, economic, and social systems, particularly in LMICs. By leveraging multi-level frameworks, engaging stakeholders, and fostering continuous learning, it is possible to enhance the translation of research into effective, sustainable health policies.

## Introduction

The implementation of sound scientific evidence has an estimated lag of 17 years from research to clinical practice or policy (1). In order to accelerate the use of evidence-based practices in real-world settings, healthcare researchers, practitioners, and policymakers are increasingly aware of the key role of implementation science in reducing the gap between what has been shown in research to be effective and what is actually practiced in healthcare, the so-called “know-do gap.” To address this gap, implementation research examines the methods and strategies to effectively integrate evidence-based practices, interventions, or policies into real-world settings. In contrast, health policy research focuses on policies’ development, analysis, and impact, often assessing their implementation and effectiveness in achieving desired outcomes.

A wealth of literature covers both implementation research and policy implementation independently (2). However, little is written about their intersection, and there has been little knowledge exchange and cross-fertilization between the two fields. Literature reviews and research overviews in each area rarely reference publications from the other, highlighting the limited integration of insights across disciplines. Moreover, even though implementation science studies have been increasingly funded in the last decades by international agencies, research evidence still plays a suboptimal role in policymaking. Of 146 studies funded by the NIH through Dissemination and Implementation (D&I) funding announcements from 2007 to 2014, 12 (8.2%) were policy studies that assessed policy content, policy development processes, or health outcomes of policies, representing 10.5% of NIH D&I funding (3). Differences in the language used by implementation researchers and policy researchers do not help. For policy researchers, policy implementation outcomes typically refer to the health outcomes observed in the target population rather than actual implementation process indicators in the implementation research language. Similarly, Implementation scientists employ the term fidelity to refer to the degree to which the different components of a given intervention, practice of policy, are implemented according to plan (4) whereas policy researchers describe it as compliance. Additionally, while Implementation science uses terms such as outer setting, outer context, or external context to describe influences beyond the implementing organization, policy research instead refers to policy fields, which are networks of agencies responsible for implementing policies and programs.

Despite growing recognition of the importance of integrating implementation science and health policy research, several factors have impeded this convergence. These include disciplinary silos reinforced by distinct publication venues, professional communities, and funding streams; differences in terminology (e.g., ‘fidelity’ vs. ‘compliance’; ‘outer setting’ vs. ‘policy field’); and divergent timeframes — researchers operate on multi-year project cycles while policymakers respond to immediate political windows. This review synthesizes existing conceptual and empirical work at this intersection, with the aim of identifying actionable pathways toward integration.

Implementation science cannot fulfill its purpose without research-driven changes in policy and practice. Conversely, policy should be informed by rigorous research to be effective. Political science and public administration have consistently urged clinical researchers to move beyond just assessing a policy's impact on health outcomes and instead actively investigate how policy and political dynamics shape the delivery of health services (5). This article presents a narrative and conceptual review of the literature at the intersection of implementation science and health policy research. It synthesizes theoretical frameworks, empirical observations, and applied experiences to identify gaps and propose integrative directions for future research and policymaking. In addition, it will provide a global south perspective since most of the studies addressing the interface between implementation science and policymaking come from high-income countries (6, 7).

Rather than applying a single pre-specified theoretical lens, this review adopts a pluralistic approach, drawing on theories, models, and frameworks from both implementation science and policy research to map areas of convergence and identify opportunities for integration.

A Global South perspective is necessary because IS and policy frameworks have been largely developed and tested in high-income countries. Factors such as fragmented health systems, political instability, donor dependency, and limited research infrastructure in LMICs create distinct barriers and opportunities that require explicit attention.

In this conceptual review, we first discuss the role of scientific evidence in implementation and policymaking. We then compare IS and PR across scope, setting, actors, and theoretical frameworks (Tables 1–3). Next, we address the interface between researchers and policymakers and the potential for integrated frameworks. Finally, we examine specific challenges and opportunities for integrating IS into policymaking in LMICs (Table 4), before drawing conclusions and proposing directions for future research.

This article intends to make three contributions: (1) a conceptual synthesis of IS and health policy research, highlighting shared goals and distinct theoretical traditions; (2) identification of barriers to integration and discussion of hybrid approaches linking implementation and policy processes; and (3) a Global South perspective that foregrounds how LMIC political and institutional realities require context-sensitive frameworks.

## The role of scientific evidence in implementation science and policymaking

**Figure 1:**
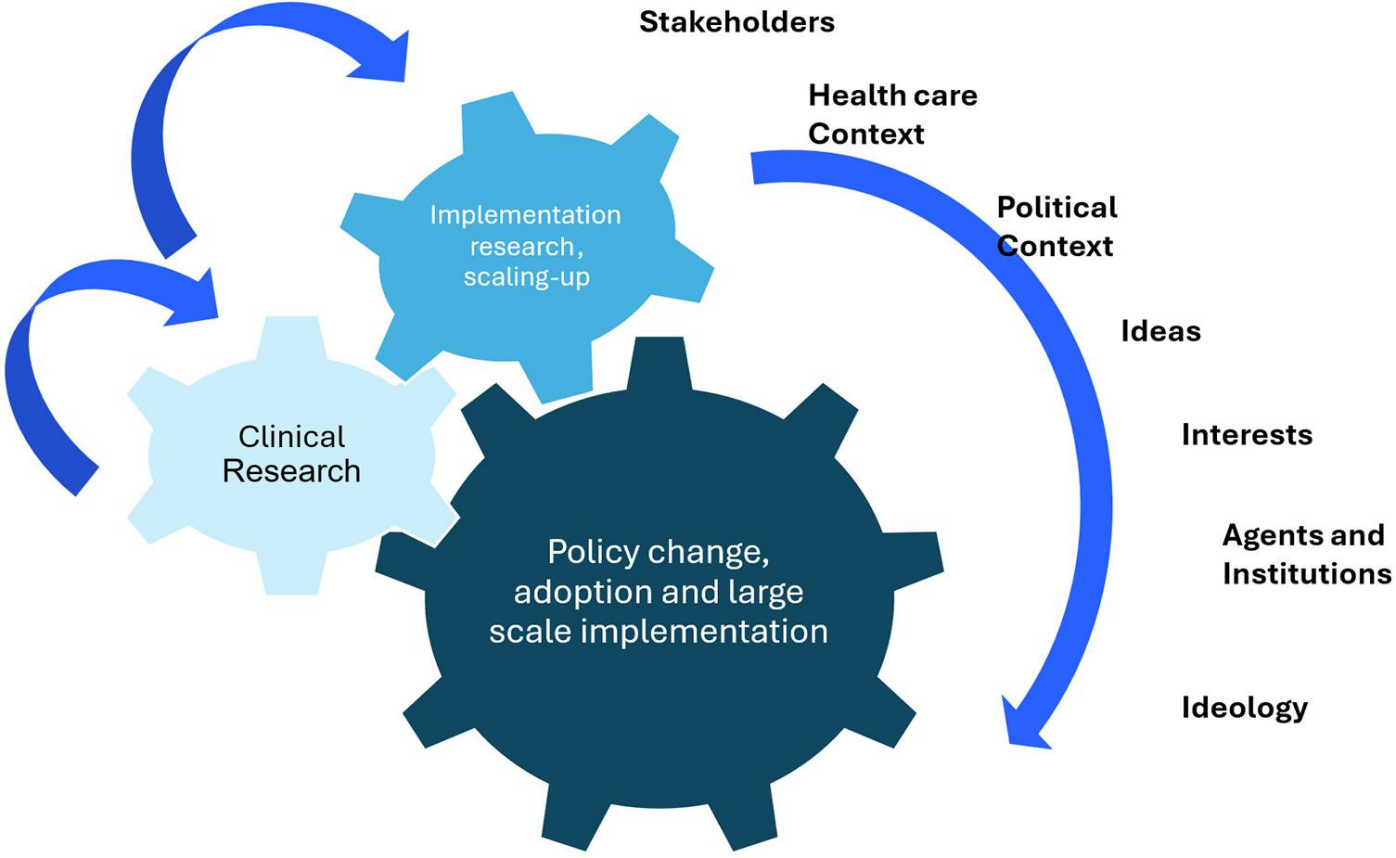
Implementation cycle: translating research into policy action. From evidence to politics.

Many clinical researchers often assume that publishing in high-impact journals translates knowledge into policy and practice through a natural diffusion process. According to this view, research impact is built on identifying the research question, elaborating a methodology, collecting and analyzing data, interpreting findings, and finally, producing policy or practice recommendations to policymakers. This “paternalistic” model is owned and controlled by researchers, who then disseminate their work to supposedly influence those to make policy decisions. Nevertheless, this assumption is not only incorrect but also naïve, and is challenged by implementation research, which promotes meaningful stakeholder involvement from the very beginning of the research process. Although this step is critical, this may still not be enough since it usually requires the elaboration of a political strategy. Scientific evidence alone does not usually drive policy changes. Even when the evidence crosses many barriers, most researchers may still think that science is the main determinant of change in policies and practices. In this regard, “contextual or colloquial” evidence such as stakeholder engagement, health care, and political context, prevailing ideas, agents and institutions, interests, and also ideologies and fashions, is equally or even more influential than the science behind the decision-making process (8). The influence of evidence varies depending on the level at which decisions are made. While clinical decisions follow a more linear evidence-to-practice model, beyond the barriers from efficacy found in classic randomized controlled trials to intervention effectiveness in the real world, decisions regarding healthcare organizations or services involve more complex dynamics, have a weaker relationship with scientific evidence, and are very sensitive to the influence and power of the key actors. At the policy level, scientific evidence plays a lesser role, with power relations, ideologies, and financial interests exerting significant influence. Of course, the stronger the evidence base and the bigger the political skills of researchers, the greater the chance of moving scientific evidence into adopting actionable policies. However, this is only an intermediate step in a broader process since adoption does not necessarily translate into implementation, as seen in the Figure 1 below.

Evidence influences policy primarily through indirect pathways — shaping the beliefs and mental models of policy actors over time, providing legitimacy to advocacy coalitions, and creating windows of opportunity when political conditions align with available solutions, consistent with the Multiple Streams Framework (9).

The figure depicts a dynamic, iterative cycle in which Clinical Research feeds into Implementation Research (scaling-up), which in turn drives Policy Change, adoption, and large-scale implementation. The three interlocking gears represent the interdependence of these processes: progress in one domain is conditional on engagement with the others. The outer ring illustrates the contextual forces shaping the entire cycle — Stakeholders, Health Care Context, Political Context, Ideas, Interests, Agents and Institutions, and Ideology — reflecting that evidence-to-policy translation is never linear but embedded in complex socio-political environments.

## Scope, setting and implementers in implementation science (IS) and policy research (PR)

IS and PR share similarities in promoting the systematic uptake of research to improve population health, but their scopes differ. In IS, the focus of implementation is often specific practices, such as interventions for hypertension or diabetes control, antenatal care, or HIV treatment, that have been proven effective through rigorous research. In contrast, PR centers on policies, supported by laws and regulations, that politicians and policymakers develop. IS usually focuses on the community, healthcare practitioners, and patients, while PR targets governmental entities and citizens. The implementation setting also varies: IS operates within defined healthcare, school or community settings, whereas PR occurs within more complex socio-political environments. In both fields, implementers are responsible for operationalizing changes such as healthcare workers in IS and government or agency staff in PR. Contextual factors beyond the control of implementers also shape outcomes, underscoring the importance of tailoring strategies to each environment (2).

Beyond these categorical differences, the comparison reveals a fundamental tension in how ‘context’ is conceptualized. In IS, context is operationalized as a set of measurable determinants — organizational readiness, inner and outer setting characteristics — that can be assessed and acted upon at the program level. In PR, context is constitutive: political, institutional, and economic structures actively shape what policies are possible, who the legitimate actors are, and what counts as a successful outcome. This distinction has direct implications for the design of integrated frameworks, which must accommodate both the bounded settings of IS and the diffuse, multi-actor environments of PR.

## Navigating the relationship between researchers and policymakers: a tale of two worlds

Researchers and policymakers operate in distinct environments, often leading to communication challenges. Researchers do not always address the specific questions decision-makers ask and may fail to report results in an accessible way for policymakers. Often, policymakers are attached to bounded rationality. Bounded rationality is the idea that people make decisions based on available information, which is limited by time, resources, and cognitive ability to operate under tight timelines and resource constraints, often seeking immediate solutions and struggling with uncertainty. This is the real world of policymakers, particularly at higher levels. Most researchers aim to generate evidence that ultimately improves health outcomes, so the studies are typically designed to address scientific questions. However, these questions often differ significantly from the equally important concerns of policymakers, who, in the best of cases, focus on how scientific evidence can be applied in practice (10). While researchers are, under the paradigm of evidence-based medicine, mostly concerned with the quality and the hierarchy of evidence, policymakers are rather focused on beliefs, value judgments, and the political context (11).

Therefore, it is key for scientists to learn how to frame a particular problem or policy for policymakers in a way that considers their judgments based on their values, beliefs, and “irrational” emotions. In addition, building mutual understanding should align with policymakers’ needs and priorities. In other words, it is not just about data or research evidence but also about compelling storytelling to frame the issue to be adopted and implemented. In this regard, the question is how far scientists should go to persuade policymakers to act on their evidence.

A shortcut to committing policymakers to the research-into-policy process is through co-creation or co-production of health interventions between scientists and decision-makers. Involving them and other stakeholders in the early stages of the co-creation or co-production of intervention components can play a key role in overcoming this disconnect (12). Although important, this may not be sufficient since most of these co-creation strategies may fail to reach higher policy levels. Even though the interactions between scientists and policymakers can range from a one-way disseminator/receiver model to a more collaborative co-production approach, there is a lack of empirical comparative studies that assess which types of interactions are most effective, for what purposes, and under what conditions (11).

In short, several mechanisms have shown promise in closing this divide: (a) research brokerage and delivery units embedded within ministries of health; (b) structured policy dialogues aligned with upcoming policy windows; (c) embedded researcher partnerships within government agencies; and (d) rapid-response research platforms enabling fast-turnaround evidence synthesis when policy windows open. These mechanisms are more sustainable when formalized through institutional agreements and protected from electoral cycles.

Effective co-production models should share four characteristics: (1) early and sustained engagement, involving policymakers from the problem definition stage; (2) mutual accountability respecting both scientific rigor and political feasibility; (3) iterative feedback loops allowing design adjustments as context evolves; and (4) explicit power-sharing arrangements recognizing that researchers and policymakers bring different forms of authority and legitimacy.

## Theories, models, and frameworks (TMFs) in implementation science and policy research

Theories, models, and frameworks (TMFs) play a crucial role in both IS and PR, guiding the understanding of how interventions and policies are formulated, adopted, implemented, and sustained. However, existing TMFs do not adequately incorporate the role of health policy in dissemination and implementation research and vice versa, hindering progress in this critical field. Policy-related constructs are often underexplored or treated as secondary factors rather than integral components of the research process (5, 13). While the two fields share commonalities, their focus and application of TMFs differ significantly. Hence, the challenge is about how to develop an integrated framework that bridges the two disciplines to streamline evidence-based policy decisions.

IS aims to understand how evidence-based interventions (e.g., clinical guidelines, health programs) are adopted and sustained in real-world settings, and its TMFs help identify barriers and facilitators to implement interventions, measure implementation and health outcomes, and develop strategies to enhance uptake and sustainability. On the other hand, PR seeks to understand how policies (e.g., laws, regulations, public health programs) are formulated, adopted, and implemented within complex political and institutional systems where its TMFs focus on factors influencing decision-making, policy diffusion, agenda-setting, stakeholder interactions, and system dynamics.

Some examples are shown in Table 1.

**Table 1:** Comparison of theories, models, and frameworks in implementation science and policy research.

Features	Implementation science TMFs	Policy research TMFs
Primary focus	Adoption and implementation of evidence-based interventions	Development, adoption, and implementation of policies
Key actors	Healthcare providers, organizations, patients, community members	Policymakers, health administrators, advocacy groups, politicians
Main outcomes	Implementation effectiveness, fidelity, sustainability	Policy adoption, enforcement, impact
Influencing factors	Organizational readiness, intervention complexity, external policies	Political will, policy windows, institutional constraints
Examples	CFIR, EPIS, COM-B, RE-AIM	Multiple Streams Framework, Punctuated Equilibrium Theory, Advocacy Coalition Framework

Conceptual advancements in these areas have expanded at an accelerated pace, leading to a growing emphasis on categorizing and organizing the numerous frameworks, models, and theories, as well as providing guidance on their application. At the same time, empirical research has also expanded rapidly, with more than 6,200 systematic reviews focusing on implementation strategies targeting consumers, providers, and organizations within the health sector alone (13). This long list of different TMFs and implementation strategies in each field creates a conundrum that makes understanding and application very challenging. Despite their differences, there are also similarities, such as the fact that both approaches have multi-level influences at the individual, organizational, and system levels, both emphasize the role of stakeholder engagement in shaping and driving implementation, and other factors such as political, social, and economic contexts influence both policy adoption and implementation of health interventions.

A brief description of commonly used TMF frameworks in IS and PR is shown in Tables 2, 3.

**Table 2:** Implementation science frameworks.

Framework	Focus	Key components	Application
CFIR (Consolidated Framework for Implementation Research) (14)	Identifies implementation determinants across five domains: intervention, outer/inner setting, individuals, and process	Intervention, Inner Setting, Outer Setting, Individual Characteristics, Process	Used to assess and tailor implementation strategies in healthcare and organizational change
EPIS (exploration, preparation, implementation, sustainment) (15)	Examines stages of implementation across different contexts to guide adaptation and sustainability	Exploration, Preparation, Implementation, Sustainment (EPIS) Stages	Guides phased implementation of programs in diverse service systems
COM-B (capability, opportunity, motivation–behavior) (16)	Explains behavior change as influenced by capability, opportunity, and motivation	Capability, Opportunity, Motivation (COM) and their role in behavior change	Used to design behavior change interventions in health and policy implementation
RE-AIM (reach, effectiveness, adoption, implementation, maintenance) (17, 18)	Evaluates implementation outcomes based on reach, effectiveness, adoption, implementation, and maintenance	Reach, Effectiveness, Adoption, Implementation, Maintenance (RE-AIM) outcomes	Evaluates the impact and sustainability of interventions in real-world settings

CFIR is particularly useful for identifying inner-setting barriers in health organizations — such as leadership engagement and implementation climate — that must be addressed before evidence-based interventions can be adopted. RE-AIM adds value at the population level by systematically assessing reach and sustainability, enabling scale-up decisions grounded in real-world evidence rather than trial efficacy alone.

**Table 3:** Policy research frameworks.

Framework	Focus	Key components	Application
Multiple streams framework (MSF) (9)	Explains how policies are shaped by three streams and windows of opportunity	Problem Stream, Policy Stream, Politics Stream, Policy Window	Explains why some policies gain attention and how decisions are made in complex government systems
Punctuated equilibrium theory (PET) (19)	Describes long-term policy stability with occasional bursts of major change	Policy Equilibrium, Punctuations (Sudden Changes), Institutional Structures	Accounts for periods of inertia followed by rapid policy change due to external or internal shocks
Advocacy coalition framework (ACF) (20)	Analyzes how groups with shared beliefs compete to influence policy over time	Advocacy Coalitions, Belief Systems, Policy Learning, External Shocks	Examines how durable coalitions shape policy processes and how learning occurs over time
Campos and Reich Framework (21)	Explores political challenges of health policy implementation through stakeholder management in six directions	Interest groups, Bureaucrats, Financial decision makers, Political leaders, Beneficiaries, External actors	Provides practical guidance for managing political, institutional, and stakeholder-related barriers to implementation

The Multiple Streams Framework explains why some evidence-based proposals gain traction only at specific political moments when problem, policy, and politics streams align. The Advocacy Coalition Framework illuminates how coalitions of researchers, civil society, and clinical communities can sustain pressure for policy change over years. The Campos and Reich framework is particularly practical for LMIC contexts, offering directional guidance for managing stakeholder engagement across six distinct actor types.

## The missing link: can we develop a comprehensive framework connecting IS and PR?

Taking into account differences and communalities, some scholars advocate for an integrated framework that bridges the two disciplines, IS and PR. A unified framework could: a) recognize the interconnectedness between the implementation of interventions and policies, b) combine theories of behavior change (from implementation science) with policy process models (from policy research), and c) address both micro or little p- (organizational) and macro- big P (policy) level factors influencing implementation success.

Bullock et al. (13) developed an integrated theoretical framework of the implementation process from a policy perspective by combining findings from the public policy, implementation science, and knowledge translation fields, exploring how policy considerations were described in implementation theories, frameworks, and processes from existing published and gray literature. It identified six ways policy interacts with implementation science and emphasizes the role of policy actors. The study presents a two-part framework: a process model outlining implementation stages and a determinants framework highlighting policy-related influences.

Some other potential approaches to integration may be the development of hybrid models, building upon already existing frameworks in the field of IS and expanding them by incorporating policy-relevant constructs and integrating policy process theories. These hybrid models can merge key components of implementation science (e.g., core determinants, implementation strategies) with policy research (e.g., political feasibility, governance), by using systems thinking. This approach would apply complex systems approaches to capture the interplay between policy formulation, implementation strategies, and real-world adoption. Developing an integrated framework could enhance the translation of policies into practice and improve the effectiveness of policy-driven interventions, ultimately bridging the gap between evidence generation and real-world impact.

IS-PR integration can be pursued through three complementary mechanisms: (1) hybrid research designs combining implementation trials with prospective policy tracking; (2) multi-level stakeholder platforms convening researchers, policymakers, health system managers, and community representatives throughout the policy cycle; and (3) systems science methods capturing feedback between implementation fidelity and policy stability. Integration may also entail risks: tensions between scientific rigor and political adaptability, and the potential for proximity to policymakers to compromise researcher independence.

## Integrating implementation science into policymaking in LMICs: some key considerations

Health policymaking in low- and middle-income countries (LMICs) often faces unique challenges, including resource constraints, fragmented health systems, and political instability. While implementation science is progressively being applied in high-income countries (HICs) to enhance the uptake and sustainability of evidence-based interventions, its integration into policymaking in LMICs remains limited. Addressing this gap requires an approach that accounts for the distinct political, economic, and social contexts. In this regard, implementation research in LMIC has primarily examined the effectiveness of strategies for implementing evidence-based health interventions rather than moving further to adoption and implementation at a larger scale.

A pivotal paper examined the characteristics of implementation research efforts in LMICs by analyzing how key implementation research principles and concepts have been utilized in published health research between 1998 and 2016 (22). The authors showed that few studies have focused on implementation strategies, reported on implementation variables, and analyzed the context under which implementation occurs. The study highlights a significant discrepancy between the theoretical principles of implementation research and their practical application, particularly concerning the large-scale impact and sustainability of health interventions in these settings. Moreover, to our knowledge, no studies have explored integrating implementation science approaches into policymaking.

It is important to note that ‘LMICs’ is not a homogeneous category. Countries differ substantially in institutional arrangements, political regimes, decentralization models, health system structures, and implementation capacity. Federal middle-income countries with established research institutions (e.g., Argentina, South Africa) differ meaningfully from lower-income, more centralized settings with limited research infrastructure. The limited evidence on IS-PR integration in LMICs partly reflects these structural constraints and partly mirrors the global skew in IS publications toward high-income settings.

The following considerations outline how implementation science could be effectively embedded within policymaking processes to drive sustainable policy change in LMICs.

### Contextual and structural adaptation

One of the primary challenges in LMICs is the fragmentation of health systems, where multiple sectors—public, social security, private, and donor-funded programs—operate independently, leading to inconsistencies in service delivery. Implementation science can help by offering systematic strategies that align policy decisions across these sectors. Additionally, many LMICs have decentralized governance structures, meaning that policies are formulated at the national level but implemented at regional or local levels. Implementation science approaches can help identify multi-level barriers and facilitators, ensuring policies adapt to diverse local contexts.

The nature and depth of decentralization varies considerably — from administrative deconcentration (where subnational units execute centrally determined policies) to fiscal and political decentralization (where subnational governments have substantial autonomy over health budgets and priorities). In contexts of deeper decentralization, national policies require significant adaptation before local implementation is feasible.

### Political economy and power dynamics

Unlike in many HICs, where policymaking is often strongly institutionalized, in LMICs, policies are frequently shaped by changing political cycles, vested interests, and informal networks. International donors and organizations also play a significant role in influencing national health agendas. Political economy analysis (23) and Advocacy Coalition Framework (20) can be valuable tools for mapping key stakeholders, power imbalances, and incentives that drive policy adoption or resistance. Successful integration of implementation science into policymaking requires a deep understanding of political realities to anticipate potential support and opposition and build coalitions for reform.

Operationally, Political Economy Analysis involves stakeholder mapping, power distribution assessment, and analysis of formal and informal rules governing actor interactions. The Advocacy Coalition Framework contributes by tracking how belief-based coalitions form and evolve, and how policy learning from implementation failures can shift coalition positions. Both tools have been applied in LMIC settings through participatory workshops facilitated by intermediary organizations with cross-sector credibility.

### Bridging the research-policy gap

A persistent barrier in LMICs is the very weak linkage between researchers and policymakers, resulting in limited use of local evidence to guide policy decisions. LMICs often lack formal mechanisms for research-policy dialogue. Implementation science can help address this by promoting co-production and co-creation models to design health interventions or policies, where researchers and policymakers collaborate from the outset to define research questions and implementation strategies, as was described above. Embedded research, a collaborative approach that integrates research into policymaking, decision-making, and implementation, can further contribute to institutionalizing evidence-informed policymaking by integrating implementation scientists into government agencies (24).

### Strengthening data and monitoring systems

Many LMICs have weak health information systems, limiting their ability to track policy implementation and outcomes. Unlike in HICs, where data-driven decision-making is common, policymakers in LMICs often rely on short-term indicators that do not fully capture the effectiveness or sustainability of interventions. Implementation science frameworks can provide structured ways to measure implementation success over time.

Monitoring systems should be co-designed with frontline implementers to ensure that indicators are feasible within resource constraints and meaningful to local decision-makers. Overly complex systems borrowed from high-income country contexts frequently fail because they do not account for data collection capacity at the primary care level or the informal care pathways common in LMIC settings.

### Cultural and social considerations

Policies in LMICs must align with local cultural, social, and behavioral norms to ensure acceptance and uptake. Unlike in many HICs, where more formal and sound healthcare systems dominate, LMICs, particularly in lower-income countries, often rely on informal providers, traditional healers, and community networks. Implementation science offers a promising opportunity to integrate community-based participatory research approaches to ensure policies are developed with and for the communities they serve.

Culturally aligned policies share several characteristics: they are developed through consultative processes including community representatives and informal health providers; they accommodate local health-seeking norms rather than treating them as barriers; they are communicated through trusted community channels; and they are monitored against outcomes that communities themselves define as meaningful.

### Opportunities for institutionalizing implementation science

LMICs must embed implementation science within national policymaking structures to ensure long-term impact. This could involve developing national implementation science training programs to build a workforce skilled in translating research into policy and fostering regional collaborations (South-South partnerships) to facilitate knowledge-sharing across LMICs.

Institutionalization requires explicit attention to incentive structures at three levels: individual (career recognition for knowledge translation outputs alongside academic publications); organizational (formalized partnerships with health ministries through memoranda of understanding); and system (alignment of funder reporting cycles with policy timelines). Without attention to these structures, institutionalization risks remaining aspirational.

Some examples of use of IS and PR frameworks in LMICs are shown in Table 4.

**Table 4:** Examples of frameworks for implementation science and health policymaking in LMIC.

Framework	Application in LMICs	Example
CFIR (25)	Identifying multi-level barriers and facilitators to policy implementation	Used in South Africa to improve HIV/AIDS treatment policies
RE-AIM (26)	Designing and evaluating scalable interventions for policy impact	Used in Argentina to improve hypertension control in the community setting
Political economy analysis (PEA) (27)	Understanding power dynamics in policy implementation	Used in Afghanistan to examine political economy factors influencing adoption of a health performance-based financing program
Advocacy coalition framework (ACF) (28)	Engaging stakeholders to drive policy change	Examines how advocacy coalitions operate to influence maternal and child health policies in Nigeria
Embedded research approach (24)	Institutionalizing implementation science in policy processes	Used in Ghana to improve maternal and child health policies

These examples illustrate that frameworks are not merely taxonomic tools but can actively bridge micro- and macro-level dynamics. The CFIR application in South Africa shows how identifying implementation-level determinants can inform national policy redesign; RE-AIM in Argentina demonstrates how reach and sustainability data at the community level can support scale-up decisions; and the PEA and ACF examples from Afghanistan and Nigeria illustrate that mapping power dynamics is operationally essential for anticipating resistance and building political conditions for policy adoption.

### Limitations

This review has some limitations. As a narrative and conceptual review, it does not follow a systematic search protocol and may not capture all relevant literature, particularly grey literature and non-English publications. The selection of frameworks reflects the authors’ knowledge and expertise and is not intended to be exhaustive but aligned with the purpose of this conceptual review. The depth of contextual analysis is necessarily limited in a review of this scope. Finally, our recommendations have not been empirically tested as an integrated package; future research should evaluate which combinations of IS and PR strategies produce the greatest gains in evidence-to-policy translation across different LMIC contexts.

## Conclusions

This review makes three contributions. First, it offers a structured conceptual comparison of IS and health policy research, identifying how differences in scope, actors, settings, and theoretical frameworks have reinforced the know-do gap. Second, it synthesizes existing proposals for integration and advances the case for hybrid models operating across micro- and macro-levels. Third, it provides a Global South-focused analysis of the structural, political, and cultural factors conditioning IS-PR integration in LMICs, drawing on illustrative cases across four continents.

Bridging the divide between implementation science and policy research is essential for translating evidence-based interventions into effective health policies, particularly in LMICs. This integration addresses the “know-do gap,” ensuring that scientific findings inform policy decisions and, conversely, that policies are grounded in robust evidence. Working with hybrid models and making implementation researchers and policymakers learn about each other's concepts and perspectives may help improve their communication skills and achieve their common goals.

In LMICs, there are additional challenges such as resource constraints, fragmented health systems, and political instability, all of which necessitate a tailored approach. In this regard, institutionalizing IS within national policymaking processes is crucial. This involves developing training programs to build local capacity, strengthening health information systems for better data collection and analysis, and promoting continuous learning and adaptation. By embedding these practices, LMICs can enhance the scalability and sustainability of health interventions, ultimately improving population health.

Several directions for future research emerge: (1) empirical evaluation of researcher-policymaker interface mechanisms across LMIC governance contexts; (2) development and validation of hybrid IS-PR frameworks integrating political economy analysis; (3) research on conditions under which co-production models maintain both scientific integrity and policy relevance; and (4) longitudinal studies tracking the policy fate of implementation research findings from publication through adoption and sustained implementation.

In conclusion, the convergence of IS and PR offers a pathway to more effective health policies. By embracing a collaborative, context-sensitive approach and by formalizing these practices within national frameworks, countries can bridge the gap between research and policy, ensuring that evidence-based interventions translate into real-world health improvements.
